# Correspondence

**Published:** 1872-12

**Authors:** N. W. Williams

**Affiliations:** Florence, Italy


### CORRESPONDENCE.

FLORENCE, ITALY.
October 29, 1872.

EDITOR DENTAL REGISTER: After some delay I will make good my promise to write something for the REGISTER. What I saw of the profession on my journey may be of some interest to your readers.

In passing along one of the principal streets in London, my attention was attracted by a large show-window filled with artificial dentures. On slabs of plate-glass, suspended by cords, were rows of teeth, to the number of perhaps one-hundred sets, mostly on rubber, and poorly constructed. On either side the window, painted on glass, and standing out in bold relief were the following advertisements: "Decayed Teeth. Mr. C—— has invented a liquid enamel, which has been proved by great experience to be admirably adapted for stopping decayed teeth. Being applied without heat or pressure, it hardens directly it is inserted into the tooth, making the tooth again sound and useful."

"Mr. C——has an improved method of extracting and arranging children's teeth. His intimate knowledge of all the branches of dental surgery enabling him to perform these operations with ease and skill, the result of many years experience; teeth extracted with his newly invented instruments."

"Mr. C—— supplies his improved mineral and natural teeth, on a principal so accurately fitted, as to require no springs nor wires, from one tooth to a complete set, without extracting the old stumps, or causing the least pain; exact in color to the adjoining teeth, and so perfect in resemblance to those they substitute, that they cannot be detected by the closest observance. In all cases giving perfect satisfaction."

Comment is unnecessary; but, truly, this bold display shows that quackery exists in our profession, as well in the Old World as in the New.

There are many dentists in London, and the few I called upon impressed me very favorably. The native dentists are too modest to assume any other title than "Mr. —— dentist."

Their offices are in good taste and comfortable. In a call of but a few minutes of course I was unable to judge of their professional attainments. All treated me with much courtesy, and pressed upon me many kindnesses, that I could not accept, for want of time. All seemed to recognize the fact that their "American Cousins" are in advance of them, which flattery I accepted with as much modesty as I could command.

Both here and in Paris, also, as I have been informed, in other prominent European cities, many American dentists occupy enviable positions; and, in truth, are the only members in the profession that have attained to any eminence.

An extensive and lucrative practice may be secured in one of two ways: First, by purchasing an already well-established business; or second, by patiently waiting, and slowly building up a good business, which last, one may do as well on one side the Atlantic as the other.

A very mistaken notion that is entertained by some, is that the only requisite for securing a fine dental practice in this country is to announce one's self an American dentist. It is true that the word American is a sort of "Open Sesame" to the confidence and purse of the Europeans; yet they are a very slow people, slow to understand, slow to believe, and slower still to adapt themselves to any new modes of operations, so that it would be worse than folly for any one simply on the strength of his being an American dentist, to expect to have a paying business from the moment he opens an office in any one of the many good localities on the line of travel from America through the Continent: though he might secure a good share of the patronage of the traveling public, yet it is from the native that he must expect his best support.

N. W. Williams, D. D. S.

